# A Review on Hypothesized Metabolic Pathways on Europa and Enceladus: Space-Flight Detection Considerations

**DOI:** 10.3390/life13081726

**Published:** 2023-08-11

**Authors:** Jessica M. Weber, Theresa C. Marlin, Medha Prakash, Bronwyn L. Teece, Katherine Dzurilla, Laura M. Barge

**Affiliations:** NASA Jet Propulsion Laboratory, California Institute of Technology, Pasadena, CA 91109, USAbronwyn.teece@jpl.nasa.gov (B.L.T.); katherine.a.dzurilla@jpl.nasa.gov (K.D.); laura.m.barge@jpl.nasa.gov (L.M.B.)

**Keywords:** Enceladus, Europa, metabolism, methanogenesis, ocean world

## Abstract

Enceladus and Europa, icy moons of Saturn and Jupiter, respectively, are believed to be habitable with liquid water oceans and therefore are of interest for future life detection missions and mission concepts. With the limited data from missions to these moons, many studies have sought to better constrain these conditions. With these constraints, researchers have, based on modeling and experimental studies, hypothesized a number of possible metabolisms that could exist on Europa and Enceladus if these worlds host life. The most often hypothesized metabolisms are methanogenesis for Enceladus and methane oxidation/sulfate reduction on Europa. Here, we outline, review, and compare the best estimated conditions of each moon’s ocean. We then discuss the hypothetical metabolisms that have been suggested to be present on these moons, based on laboratory studies and Earth analogs. We also detail different detection methods that could be used to detect these hypothetical metabolic reactions and make recommendations for future research and considerations for future missions.

## 1. Introduction

Many possible ocean worlds have been identified, including Enceladus and Europa, moons of Saturn and Jupiter, respectively [1,2,3,4,5,6]. Both of these moons have been of great interest to the astrobiology community: Europa will be visited by the ESA’s JUpiter ICy moons Explorer (JUICE) and Europa Clipper [7,8,9]. The 2023–2032 Planetary Science and Astrobiology Decadal Survey included Enceladus in New Frontiers (NF) calls, and Enceladus Orbilander was the second highest prioritized as a flagship mission after Uranus Orbiter and Probe [1]. In addition, ESA’s Voyage 2050 also prioritized “Moons of the Giant Planets”, which included focus on both Enceladus and Europa [10]. Previously, NASA’s Cassini mission identified organic materials, salt, and molecular hydrogen in Enceladus’s plume that is connected to the ocean [5,6,11,12,13,14]. Both moons also display evidence for energy sources (e.g., molecular hydrogen in Enceladus’s plume, [14]); hydrogen peroxide on the surface of Europa, [15] and tidal heating (e.g., [16,17]). However, many fundamental questions remain unanswered about Enceladus and Europa, including whether these moons host life [1,18,19,20,21].

Metabolism is an essential part of life on Earth and metabolic products have been proposed as detectable and reliable biosignatures to search for on other worlds (e.g., [22]) though only if their context is well understood. The central biological process of metabolism is a series of complex chemical reactions that sustain life. While there are several proposed theories on metabolic evolution (e.g., [23,24]), the origin of metabolism is not well constrained. While no life detection mission is currently planned to Europa or Enceladus, researchers have theorized what kind of metabolisms could exist outside of Earth through different methods including experimental work and modeling (e.g., [25,26]). For the purposes of this review, we will assume an origin of life event occurred on Europa/Enceladus in regards to the metabolisms proposed. We will not be discussing origin of life type events on either world or speculating what the original organisms on these worlds could be and instead focusing on their current habitability.

In this review, we will focus on possible metabolisms in these two worlds. We will present the hypothesized ocean conditions on Enceladus and Europa and introduce the possible metabolisms that could survive under such conditions. We will also discuss different methods posited to detect such metabolic reactions. Lastly, we will introduce recommendations for future research in this field, including research and mission instrumentation development.

## 2. Hypothesized Ocean Conditions on Enceladus and Europa

The conditions and constraints of liquid water oceans on Europa and Enceladus (i.e., temperature, pressure, ice shell thickness, and salinity) need to be well understood to determine chemical availability and what kind of metabolic reactions could occur on either moon (Table 1). These conditions are derived from mission data (i.e., the Cassini mission to Saturn and Galileo mission to Jupiter) along with modeled results. There are still significant unknowns about these oceans and many of the constraints are estimated based on the modeled results. For example, life—as we know it—is based on redox cycles that would necessitate some kind of a chemical disequilibrium, or redox cycling, to be present in the ocean, with a sustained source or influx of material in order to maintain it. By understanding the ions and gases present in the oceans, we can better evaluate if such metabolisms are reasonable under those conditions.

### 2.1. Europa Ocean Physical Parameters

Icy/ocean worlds like Europa that formed in the outer solar system are thought to be more volatile-rich than the planetary bodies in the inner solar system [51] Europa’s structure is made up of an archetypical layered ice shell with a rigid, stagnant lid which overlies a convecting ice mantle that has active tectonism [52]. Europa’s ice shell thickness has been debated but is believed to be between ~5–100 km (Table 1, [37,38,39]) with a ~100 km deep ocean based on analysis of tidal heating, subduction, dissipation, and conductive cooling, e.g., [17,52,53]. There is limited evidence that water plumes may be currently erupting from Europa’s surface, allowing for a possible future opportunity to collect the ocean material and study it from space (e.g., [54,55,56]).

Irradiation of the Europan ice shell by high-energy particles likely produces strong oxidants such as a thin molecular oxygen atmosphere [57], hydrogen peroxide (H_2_O_2_), and various oxidized sulfur species like SO_4_^2−^ [15,58]. The top few meters of the ice shell probably contain a strong chemical gradient from regolith alteration processes related to, by vacuum, exogenous materials, Jupiter’s radiation, and potential plumes reaching the surface [54,55,56,59,60]. The water–ice and water–rock interfaces are critical sites for creating chemical disequilibrium with steep local osmotic and ionic gradients [61].

Oxidants on the surface could be transported to the oceans where they would encounter reductants, thus altering the chemistry of the ocean, which may in turn increase the habitability potential of the oceans [15,44,62]. The potential rates of transport for oxidants from the ice shell to the interior ocean are strongly dependent on the composition of the ice shell, as the presence of concentrated impurities can lower the melting temperature of the ice [63], aiding in transport to the ice–water interface which is probably isothermal [18]. Some researchers [64] suggested that impacts on the ice shell could have created large melt chambers occurring near the surface, which could generate a continuous surface-to-ocean melt column. This melt drainage could provide a consistent means of exchange between the surface and ocean. There are several possible vertical transport processes such as subduction, brine drainage, and penetrating impacts [64].

Europan surface images show morphologies consistent with formation by extensional tectonics [65,66], and changes within band morphologies suggest formation styles similar to mid-ocean ridges on Earth [65,67,68]. Tidal models suggest that energy dissipation primarily occurs in the ocean which may cause outer shell flexing but probably has a negligible impact on the interior [20]. Although some of the tectonic processes on Europa may be similar to Earth’s, the major thermal activity on the Europan seafloor is quite distinct from Earth. The processes warming Europa’s ocean floor are more likely a product of passive cooling of radiogenic heating of the solid interior [34]. These processes may cause thermal contraction within the ocean floor, leading to cracking, which creates a pathway for fluids to penetrate the seafloor [34]. In this scenario, the fluids would generate reduced hydrogen from serpentinization equaling or exceeding potential fluxes from high-temperature hydrothermal activity [34]. The tidal heating that warms Europa is expected to create prolonged water–rock interactions comparable to some hydrothermal systems on Earth [33]; this heat source for seafloor hydrothermal environments is preferred over active volcanism which is unlikely based on modeling of accretion and cooling [68]. Reactions of hydrothermal systems increase the likelihood of a habitable Europa, and hydrothermal systems on Earth are potential sites for the emergence of life (e.g., [69,70,71,72,73]).

### 2.2. Europa Ocean Composition

Europa’s ocean is poorly constrained, but proposed to contain a number of ions, dependent on planetary and crustal formation [74]. It is unknown how much oxygen is transported to the ocean, and what abundances of oxidants are required to generate and maintain redox gradients within Europa’s ocean [15,44,62,64]. However, there is also believed to be diffusion transport for these materials and heat [74,75,76]. For example, a large quantity of exogenic sulfate may be supplied into the ocean from the icy crust, e.g, [77,78].

Many geochemical models of Europa’s ocean chemistry used surface and atmospheric observations to ascertain what main ionic species are present. These early proposals for ocean chemistry considered three primary options: a neutral Na–Mg–SO_4_–H_2_ solution, an alkaline Na–SO_4_–CO_3_ solution, or an acidic Na–H–Mg–SO_4_ solution [74,79,80]. Zolotov and Shock [47] developed a model informed by Brown’s [81] Earth-based observations of chemical species detected in the Europan atmosphere, suggesting that Europa’s ocean composition was generally comparable to Earth’s, with SO_4_^2−^, Mg^2+^, Na^+^, and Cl^−^ as the dominant species. Other geochemical models recognize similar dominant species, but the relative abundance (NaCl-dominated vs. MgSO_4_-dominated) is not generally agreed upon [30,43,44,45,46,47,48,49,50,74,82,83,84]. The discrepancy between the models is because some models predict that the original composition of Europa’s core would create the Mg- and S-rich ocean mentioned above (e.g., [74,82]), while other models predict that water–rock interaction at a silicate-rich seafloor would generate a Cl-rich ocean.

In terms of pressure, the near-vacuum conditions at the surface of icy moons like Enceladus and Europa become irrelevant at relatively shallow depths, well below the hypothesized ice shell thicknesses of Europa and Enceladus [83]. For example, Na^+^ and Cl^−^ ions have been detected in Europa’s atmosphere by the Galileo spacecraft [85,86]. These detections of chloride salts on Europa support that Na^+^, Mg^2+^, and Cl^−^ could be the major constituents of Europa’s ocean, consistent with the models mentioned above (e.g., [47]), although the hydration states of these salts are debated upon. For example, Journaux et al. [87] argue that there is separation within the ice shell caused by hyperhydration states of salts. Previously identified NaCl·2H_2_O in the lower convective ice crust, and 2NaCl·17H_2_O present within the top few kms of the ice crust, would allow for ionic exchange. Furthermore, the presence of a chloride-rich ocean [43,45,77,78] may not be consistent with extensive inputs of exogenic sulfate into the subsurface ocean [77]. In addition, Melwani Daswani et al. [30] developed a model which highlights that a H_2_CO_3_ (carbonate)-dominated ocean is also possible.

Potential indicators of ocean composition include the presence of MgCl_2_ and NaCl in chaos terrains (geologic features found on the surface of Europa) on the surface [29,43]. However, because the rest of the exact salt inventory is unclear, a lot of research substitutes salts that are precipitated from elements that are present within carbonaceous chondrites (e.g., [88,89]), as well as those theoretically understood to be thermodynamically feasible at the Europan surface (e.g., [47]). Ocean circulation, geological activity (e.g., hypothetical plumes [54,55]) and thermal history will have resulted in global salt transport [90]; because of this, habitable environments on Europa are thought to be primarily saline. Salinity estimates for the ocean vary widely, with an estimate of 50 ppt at the lower end [91].

Sulfur (partly bonded with O) was detected on Europa’s surface [92,93] and SO_4_^2−^ was detected in the non-ice surface material [44]. Observations from ground-based telescopes show evidence of sulfuric acid hydrates accumulated in Europa’s trailing hemisphere [29,48,77]. These hydrates are thought to originally be present on Io as sulfur ions and sulfur dioxide, and then be later oxidized into sulfuric acid hydrates, creating a potential consistent source for Europan sulfur over long time periods [29]. Models of the development of Europa’s ocean could provide a basis for the ocean composition—recent modeled efforts indicate that if the ocean was derived from thermal evolution, it would have been carbon- and sulfur-rich [30].

Hydrated sulfates are found in chaos terrains, suggesting that the sulfur is coming from an underlying ocean [43,44,46,47,48] and delivered from Io [50,62]. These data have led to the wide adoption that Europa’s subsurface ocean likely contains sulfate [29,46,74,94]. The presence of sulfates in Europa’s ocean would have required escape of H_2_ [82,95]. With those assumptions, Zolotov and Kargel [82] suggested that Europa’s ocean would contain sulfate, Mg^2+^, Na^+^, and Cl^−^ as major solutes, overlying rock made of phyllosilicates (serpentine, saponite, chlorite), chromite, andradite, carbonates (calcite), Fe-sulfides (pyrite), and organic compounds. Zolotov and Shock [95] advocate indigenous abiotic sources of organic compounds such as Fischer–Tropsch–type (FTT) synthesis from cooling volcanic gases.

In regards to evaluating the pH of the Europan ocean, assumptions are either made about surface material interfacing with the ocean or by understanding the core. The pH is strongly dependent on the rock composition of Europa’s seafloor [29,30]. The degassing of sulfur dioxide and carbon dioxide would form sulfuric and carbonic acid and most likely make the ocean floor of Europa very acidic and reducing. In addition, pHs as low as 2.6 have been hypothesized [62]. The poorly constrained pH of Europa is further presented in Merino et al. [96] with a potential pH range for Europa of ~2–11. If the seafloor rocks are similar to carbonaceous chondrites, the fluid pH would be alkaline regardless of the existence of CO_2_ [41]. Europa is expected to have a differentiated iron-rich core and a silicate mantle [97]. It seems most likely, from the bottom up approach (due to the mass, density, and volume) that the pH of Europa would be acidic (~4–6) [29,30,62]. However, these numbers can vary and are not well constrained.

Ocean temperatures for Europa should lie close to freezing; however, salinity effects could reduce the temperatures to 250 K [37]. Moreover, despite the ocean being 100 km deep, the sea-floor pressure has been predicted to be 130–360 MPa [33,34] and more recently as low as 110 MPa because Europa’s gravity is less than one-seventh of Earth’s [37]. These pressures and temperatures dictate that the inorganic carbon stays in dissolved form [45]. As Europa formed, it likely consisted of between 5–10 wt.% ammonia [49]. However, the icy shell of Europa formed may have led to ammonia being excluded from the freezing ice, creating a surface with no ammonia with an ammonia-rich subsurface ocean [48]. It is likely that nitrogen initially existed as primarily ammonia during Europa’s formation, but nitrate would eventually become the main phase of nitrogen if Europa’s waters became oxidized.

### 2.3. Enceladus Ocean Physical Parameters

Enceladus is thought to have a thick (21–25 km) icy crust with thinner (<5 km) regions at the southern pole [32,36,40] and is believed to have relatively low ocean pressures [32,35]. This crust sits atop a large subsurface ocean that then interfaces with the rocky core [32,40,98]. The core is hypothesized to be low density based on Cassini data [32,99]. Additionally, a plume has been observed in the Tiger stripes region in the southern pole of the moon [100] which is believed to be sourced from the ocean. This plume could provide a seemingly direct view into the ocean chemistry and conditions.

Enceladus’s core is thought to be low-density and unconsolidated, with chondrite composition [16,101]. Additionally, this rocky core interacts with the subsurface ocean, and it is theorized that this creates a heterogenous structure of an outer layer rich in carbonates, and a serpentinizing inner layer [27,101,102,103]. Serpentinization is a process where water enters planetary crusts and ferromagnesian minerals are altered through metamorphism and hydration to produce hydrogen and methane [102,104,105,106,107]. The process of serpentinization has been observed within some hydrothermal systems on Earth. This process of serpentinization can abiotically create organics from the heated environment and the water–rock interfaces. Specifically, methane and hydrogen, along with the reactive iron minerals, can be useful for abiotic and biotic organic molecule synthesis. Based on interpretation of plume measurements described later, it is possible that serpentinization and hydrothermal activity could be occurring within Enceladus’s ocean floor [108]. Recent work also theorized that redox reactions occurring at the sea floor could be occurring with the presence of quartz, talc, and carbonate materials [27].

Previous studies have shown that Enceladus’s ice crust may be thermally conductive, allowing it to maintain more heat than a convective ice shell, and, if so, the equilibrium heating rate of the ocean is projected to be able to sustain a long-lived subsurface ocean environment [109,110]. Similar to Europa, the likelihood of a liquid ocean is of particular interest, as a continuous liquid environment with interactions between the icy crust and rocky seafloor provides the potential for geochemical reactions that can provide many of the building blocks necessary for life. If these interactions persist over long periods of time, the possibility of them producing biologically important reactions increases. To investigate these possibilities and gain a full picture of the environment within any subsurface ocean, investigating the ocean directly through in situ sample analysis is paramount. Enceladus provides a unique environment for the investigation of the subsurface oceans with the presence of the long-lived plume emanating from the south polar terrain, which is believed to be sourced from the ocean [5,6,111]

### 2.4. Enceladus Ocean Composition

Measurements from Cassini’s Cosmic Dust Analyzer (CDA) [112] and Ion Neutral Mass Spectrometer (INMS) [113] instruments analyzed samples from the ejecta of Enceladus’s plume providing direct insight into the liquid environment. The data from INMS and CDA indicate the plume composition was mostly water vapor and other gases such as carbon dioxide and ammonia [40]. Water particles were also found to contain 1% salt compounds (primarily NaCl) and NaHCO_3_ and/or Na_2_CO_3_ [5,6]. Molecular hydrogen was detected and is thought to be produced within the subsurface ocean as opposed to being a byproduct of plume sample fragmentation within the instrument [18]. Additionally, silica particles on the nanosized scale were detected within Saturn’s E-ring and are attributed to Enceladus’s hydrothermal activity [109]. In addition, recently, phosphorous in the form of phosphates, critical for life, was detected within the E ring, which is sourced from the plume [12]. As phosphates are critical for metabolic pathways (e.g., adenosine triphosphate (ATP), the energy currency currently used in all living cells), this recent discovery is particularly interesting and should be further evaluated when considering metabolic pathways.

Both low (<50 amu) and high (>200 amu) mass organics were also detected in the plume samples and were mostly composed of carbon, hydrogen, oxygen, and nitrogen with amines, carbonyls, and aromatic compounds present [11]. However, approximately 4% of all the materials contain complex organics primarily composed of unsaturated and aromatic nitrogen containing hydrocarbons [13]. However, these measurements of Enceladus’s plume may not be completely representative of the ocean composition, as the eruption process of the plume might have altered the composition and fractionated the plume from the original source [28].

Interpretations of Cassini data along with modeled work and laboratory studies indicate an alkaline ocean (pH ~8–9 as the current best estimate but 8.5–11 is most commonly discussed; Table 1) rich in sodium chloride and sodium bicarbonate salts [27,28]. The presence of the NaCl salts and carbonate compounds indicate that the subsurface ocean is interacting with the rocky carbonate-rich ocean floor [27,114]. Evidence from previous modeling and image analysis studies suggests a warmer ocean [16,109,115].

Silica [108], H_2_ [14], and the organics (e.g., [11,13]) present within the subsurface ocean on Enceladus point to potential hydrothermal activity, which could possibly include a hydrothermal vent system [108]. The presence of these compounds in contact with a rocky ocean floor with a high pH environment implies that serpentinization is occurring within Enceladus [102,114] through water–rock interactions within the chondritic core [20,101].

## 3. Proposed Metabolisms

**Figure 1:**
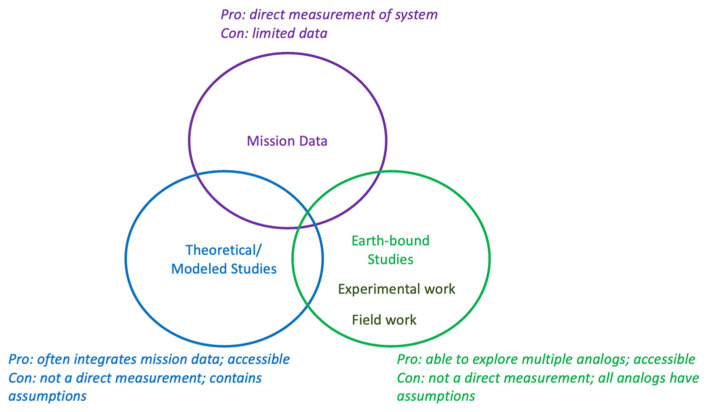
Types of research discussed here for hypothesized metabolism.

Due to their liquid water oceans and potentially habitable conditions, both Europa and Enceladus are attractive targets for astrobiology. Therefore, significant research has been conducted to understand possible metabolic/biotic processes on these worlds. The research on metabolic processes falls broadly into three key categories: inferred from direct observations, modeled/thermodynamic experiments, and research using Earth-based ocean world analogs (Figure 1). The modeled data are often directly related to mission data as well. Analog work can involve doing field sampling of different environments on Earth as well as trying to replicate different conditions to a laboratory setting. In addition, field work can utilize microorganisms and directly observe their behavior within ocean world-relevant environments. Overall, all of these modes of observation are important for understanding the possibility of life on these moons.

As an important note, this review is specifically focused on terrestrial life/metabolic pathways. As the biosphere on Earth is our best understanding of life in the Solar System, this is a logical starting point. There may be extraterrestrial metabolisms that are not known or understood.

### 3.1. Europa

The radiolysis occurring on Europa by magnetospheric ions from the Jovian system and the water ice within the surface ice present a potential source of molecular oxygen to the subsurface ocean [116,117]. If this oxygen can pass from the surface of Europa to the crust–subsurface ocean interface, then its presence could enable aerobic respiration (respiration requiring oxygen) to occur in Europa’s subsurface ocean [45]. Aerobic respiration is energetically favorable within metabolisms on Earth, and the oxygen is able to produce significantly more ATP compared to anaerobic respiration; while the presence of molecular oxygen does not guarantee aerobic respiration, it introduces the possibility [26]. However, given Europa’s possibly thick ice shell (Table 1), the diffusion of molecular oxygen to the subsurface ocean is not guaranteed. Additionally, for redox chemistry to occur, reductants would have to be present at the crust–ocean or ocean–ice interface. As their most likely source is the rocky mantle, their presence at the crust–ocean interface would suggest the need for a high degree of mixing within the ocean, which may not be the case. Thus, anoxic metabolisms, or metabolisms without oxygen, must be considered as well [118].

Understanding potential geobiological relationships in potential Europan hydrothermal systems requires comparisons of fluid compositions accompanied by the analyses of Earth-bound microbial communities commonly present in these environments. Many distinct metabolic pathways in the Europan ocean have been considered in the literature (e.g., [62,119,120,121,122] in addition to the most likely organisms to inhabit these environments [123]). The well-known redox pairing of anaerobic methane oxidation and sulfate reduction, widely present on Earth’s ocean floor, has also been proposed for Europa [124]. Some genomic studies have been carried out identifying potential candidate species on Earth that are not dependent on nitrate/nitrite for metabolism, but they are as of yet uncultured [125].

Early studies into potential Europan life examined the likelihood that photosynthesis could be a viable metabolism (e.g., [126]). Photosynthesis on the surface of the ice shell was discounted as the temperature is too cold for life and because of the irradiation. In the subsurface ocean, light could not penetrate the thick ice shell to the ocean, and photoautotrophy based solely on light emitted from black smokers is not likely (e.g., [127]). On worlds where light is not a possible energy source, natural selection would likely favor the evolution of alternative cellular carbon fixation mechanisms [62]. Gaidos et al. [120] argued that geochemical cycling under Europan conditions might not support life using metabolic processes that we know of. Instead, life needs to be supported by chemical energy sources in the subsurface—such as the ocean—relying on the aforementioned transfer mechanisms for reactants from the surface or at the rock–water interface [62,95].

On Earth, extremophiles [128] including archaea and bacteria live in extreme environments such as hypersaline environments, places that have extreme temperature or pressure, and places with extreme pHs. Extremophiles are the most analogous lifeforms to potential life on Europa [96,129]. Examples of extremophiles include acidophiles (<pH 5) and hyper-acidophiles (<pH 3), found in environments like hot springs [130], and alkaliphiles (>pH 9) and hyper-alkaliphiles (>pH 11, also present in hot springs, as well as terrestrial serpentinizing systems, and are possibly more relevant to Enceladus (see below) (e.g., [131]). The literature reflects that pH is possibly the main parameter that controls the abundance and composition of microbial communities on Earth [132,133,134], and with the pH of Europa’s ocean being so poorly constrained, it is difficult to ascertain which adaptions are more likely. With a likely acidic ocean, the intersection between acidophiles and psychrophilic organisms (extremophiles that grow in colder temperatures) seems particularly relevant for Europa.

In terrestrial deep ocean hydrothermal environments, the preferred lifeforms are typically chemoautotrophs. Many of the redox compounds commonly used by chemoautotrophs have been theorized to be present in Europa’s ocean. Zolotov and Shock [43] modeled tidal heat processes and showed that if tidal heat is produced in the silicate mantle and hydrothermal systems are also present, then methanogens or sulfate-reducing organisms could exist because they could use dissolved H_2_ from serpentinization as an energy source. The radiolytically generated oxidants at the surface may represent another energy source for chemotrophy [134] when combined with reductants produced at the seafloor [34,45].

The Gakkel Ridge in the Central Arctic Ocean is a field site containing black smokers in the Aurora Vent Field (AVF) growing off a bedrock of pillow basalts and was first dived to by an ROV in 2021 [135]. Black smokers (and white smokers) are acidic high temperature vents; these are influenced by magma, unlike lower temperature alkaline vents produced by water–rock interactions. The Gakkel Ridge is significant as the hydrothermal vents are the first beneath permanent ice cover [135]. The physicochemical and microbiological characterization of the potential vent plume showed evidence for methane and possibly hydrogen-enriched vent fluids, providing enough energy to support large amounts of microbial activity in the plume [136]. The types of autotrophic microbes inhabiting hydrothermal vents are defined by fluid chemistry such as fluxes of seafloor reductants from hydrothermal activity [45].

Other subsurface analog environments exist where water and energy sources create chemical species that support deep water microbial activity [137]. In the Mponeng mine (which has low O_2_ and temperatures similar to predictions for the Europan ocean), there are chemotrophic organisms that fix their own carbon and thrive in radiolysis–induced chemical disequilibria [122,137]. On Earth, complex brines (like the ones possibly found in the Europan ocean, based off ion detections on the surface) contain many halophilic organisms [138] including organisms existing below 0 °C [139]. It is likely that Europa has a briny ocean (e.g., [89]), composed of MgSO_4_, Na_2_SO_4_, and Na_2_CO_3_ [94,140] and is possibly NH_3_/NH_4_^+^-rich [48]. If these assumptions are true and outgassing is minimal, any potential adapted organism must be tolerant to ammonia brine. The cycling of sulfur, methane, and iron in briny habitats and a lack of sunlight on Earth show that extremophiles can possess relatively flexible metabolic structures [138]. Metabolic processes in life as we know it have only generally been detected at above −20 °C, and life usually becomes dormant at lower temperatures. This is very close to the theorized temperatures of the Europan ocean, which would be close to freezing, though Thompson et al. [37] predict the salinity will probably reduce these temperatures to around 250 K (~−23.15 °C). Some authors have theorized that psychrophiles that are halotolerant might be able to persist on Europa in the saline subsurface oceans [82].

### 3.2. Enceladus

Enceladus, while similar to Europa in many aspects, contains a different range of environments and presents another interesting case for astrobiologists to consider. While the ocean has not been directly sampled, material from Enceladus’s south polar plume provides insight into its ocean’s composition and by extension, potential metabolism that could exist there. One of the most important discoveries within the plume to date has been molecular gaseous hydrogen (H_2_) [14] and organic material, including acetylene in the ice grains [11]. The discovery of the acetylene-metabolizing bacterium microbe *Pelobacter acetylenicus* on Earth offers some validity to the possibility of acetylene-metabolism occurring on Enceladus. The hydration of acetylene to acetaldehyde ultimately leads to the generation of substrates that can be used as energy sources for other relevant anaerobes (e.g., methanogens and sulfate-reducers) [141].

Methanogenesis is one of the key postulated metabolisms to be possible in Enceladus’s ocean [142,143,144] due to the molecular hydrogen (H_2_) detection in the plume of Enceladus by Cassini. Methanogens on Earth are limited in their metabolic pathways, and to date, only three pathways are known for cellular respiration [145]. While each methanogen species will have their own reactants and products associated with their biochemistry, a few products are consistently seen across the mechanisms. Major constraints on what conditions on Enceladus would be habitable for hydrogenotrophic methanogenesis are typically based on salt content, temperature, and pH [143].

The methanogenesis pathway has been detected in an ice-covered Antarctic lake with little light penetration, which can be considered an Earth analog for Enceladus conditions [146,147,148]. The anoxic portion of the lake had high portions of methane, and metagenomic profiling of samples from the anoxic zone of this lake revealed complete pathways for methanogenesis [147,148]. The detection of methanogenesis pathways in one of the best terrestrial analogs of Enceladus is promising evidence for the possibility of methanogenesis as a metabolic pathway in Enceladus’s ocean. The evidence is not limited to metagenomic analyses only; methanogenic archaea (most notably, *M. okinawensis*) have been successfully cultured under putative Enceladus conditions [149,150].

In addition, the theoretical living environment within Enceladus’s ocean was estimated to be 5 × 10^−6^–5 × 10^3^ cells/mL based on the flux of the hydrogen that has been detected within the plume related to methanogenesis [142,151]. As there is major uncertainty to the available energy of Enceladus, there is a significant range in the hypothesized biosphere. A consideration for missions is the number of cells that can be collected over different timeframes. For example, using the plume, it is hypothesized that 10^5^ and 10^8^ cells could be collected from the plumefall over 100 days, with 10^5^ cells/mL needed for detection [151]. This is, of course, dependent on mission design, sampling technique, sample storage, and extraction mechanisms. In addition, there are possible concentration mechanisms that could be occurring in the plume [151] which could make this detection easier.

Enceladus could also support aerobic metabolism. Radiolysis of icy surfaces has been discussed as a process occurring on many icy moons, including Enceladus, with the ability to produce various products such as O_2_, O_3_, and H_2_O_2_ [152]. With the deposition of the plume’s ejecta back onto the surface, the production of O_2_ from this process will be buried and subsequently have lesser interactions with the energetic particles preventing further radiolysis [21,152]. The presence of O_2_ under the ice crust of Enceladus could enable aerobic metabolism. This scenario is dependent on more than just the availability of radiolytic products, but some models suggest that aerobic metabolism may be energetically favorable on Enceladus [21]. Recently, iron reduction has been explored as a viable metabolic pathway; this has been examined through laboratory studies [153].

In addition, many of the field work analogues for ocean worlds tend to be more general (as discussed above) and can be applied to Enceladus. This is especially true as the plume containing material is proposed to be from a hydrothermal system. For Enceladus metabolisms, due to Enceladus’s pH, alkaline hydrothermal vents can be considered more applicable. For instance, Lost City, an alkaline vent system on Earth powered by serpentinization [154,155,156], contains organisms likely to consume H_2_, CH_4,_ and formate produced within the hydrothermal environment (e.g., [156]). Metabolisms utilized in Lost City include sulfate reduction, anaerobic oxidation of methane, and carbon fixation [157,158]. For example, methanosarcinacae (within high temperature areas) and anaerobic methanotrophic archaea (within cooler areas) have both been found within Lost City [158]. Additionally, sulfate-reducing species such as thermodesulfovibrionales are also present [156]. Microbial communities in these areas exist within a symbiotic environment, relying on sulfate reducers to provide CO_2_ [159,160,161]. These sulfate reducers could be the primary consumers of formate. Methanogens present within these environments would then utilize the CO_2_ as a carbon source.

## 4. Summary and Comparisons between Europa and Enceladus

While Europa and Enceladus have possible very different ocean settings and habitable conditions, the techniques used to study these worlds have commonalities. While the conditions (e.g., pH, salts/salinity) can vary, the different worlds are both believed to be habitable. The data and uncertainties for the moons also vary. With the assumptions and unknown variables on the worlds (e.g., the pH of Europa), actually constraining all of the possible metabolisms is a challenge. Europa’s ocean is especially unconstrained. However, as expected, there are differences in the key metabolisms discussed for both worlds.

Europa and Enceladus may host diverse populations with varying metabolisms that could thrive in these harsh environments. This is hypothesized based on our understanding of Earth’s biosphere. Methanogenesis [146,147,148] is the most common metabolism postulated for Enceladus primarily due to in situ detections of CH_4_ within the plume (e.g., [14]). On the other hand, likely due to the lack of direct detections and different theories about how nutrients are cycled, metabolic processes suggested for Europa are more varied but primarily relate to anerobic pathways including methane oxidation and sulfate reduction (e.g., [62,119,120,121,122]).

Understanding the origin of life on Earth and early life could be beneficial for looking for life on ocean worlds [162]. As noted, many of the field work sites relevant to ocean worlds and the exploration of extremophiles including research of the ocean floor/hydrothermal systems are broadly important to ocean worlds, and hydrothermal vents are known for hosting a variety of extremophile metabolisms [96,163]. This holds true even with the significant pH, pressure, and salinity differences between both moons. Even without constraining the ocean world conditions, better understanding of these terrestrial environments and life inhabiting those places will benefit ocean worlds investigations. In addition, this research is often conducted outside of the planetary science/astrobiology communities, making interdisciplinary collaboration critical to this work. However, it is important to consider the caveat of these studies with the parameters of the oceans on Europa and Enceladus. In addition, hydrothermal systems are localized environments and would be difficult to analyze with current mission technology. Therefore, this review focused more on metabolisms that are suggested from a bulk ocean composition.

Overall, better constraints for both worlds (including geophysical characterization of the ocean and inventory of the organic/inorganic materials available) would greatly improve the understanding of habitability and what metabolisms are possible. Therefore, future missions to these worlds are imperative.

## 5. Implications and Recommendations

Based on this review, recommendations for future work are tabulated in Table 2.

Our knowledge of the conditions of Enceladus and Europa is very limited and such conditions are very important values (pH, pressure, salts, temperature, water activity, oxidants) to constrain what types of abiotic and biotic processes are plausible. Especially critical to this is constraining the salt concentration and composition on these worlds. As there is currently a range of pHs and salts on these ocean worlds, we recommend varying these conditions for biotic and abiotic studies.

In order to truly understand the conditions on these worlds, more missions to ocean worlds are required. These laboratory and analog studies can then be connected to future mission data. Europa Clipper and JUICE [7,8,9] will provide much needed information about Europa’s habitability, and allow for better modeling and understanding of the ocean conditions (Table 2). Proposed Enceladus NF mission concepts as well as the Orbilander flagship [164] would then provide constraints for Enceladus. Flown missions to these worlds would perhaps be the most critical addition to our knowledge about the oceans on these worlds as well as any possible life that inhabits them. Future missions to Enceladus to both better characterize the ocean and look for life would be especially important for understanding this. Funding a mission to Enceladus would be critical to not only searching for life, but to constrain and better understand what life could inhabit planetary systems.

In these missions, searching for the precursors and products of these metabolic processes as well as complex organic molecules would answer questions about these metabolisms. In particular, constraining the hydrogen and methane on Enceladus further and understanding how these values change over time would be an interesting data point to consider. In regards to Europa, constrains on the ocean conditions must be made in order to constrain what is best to look for. For these molecules, mass spectrometry would be a viable technique (both for volatiles and ice grains). In addition, there are many other factors to understanding the viability of possible metabolisms, including the presence of trace metals, cofactors, and enzymes or other biological macromolecules.

Experimental studies of abiotic reactions will also help constrain the materials that have been observed on Europa and Enceladus, specifically to generate the chemical signatures in line with the metabolisms proposed. By understanding what abiotic chemistry is capable of, researchers will be able to better differentiate biotic and abiotic processes [176]. Work to understand the abiotic generation of methane/hydrogen under Enceladus conditions, for example (e.g., as conducted looking at Mars [165]), could be directly compared to the plume conditions to help constrain possible biotic/abiotic processes. We specifically recommend focusing on methanogenesis for Enceladus and methane oxidation and sulfate reduction for Europa for these abiotic studies. Exploring both lab work and modeling work in parallel is also recommended. This is especially critical to explore under relevant geologic conditions, such as serpentinization for Enceladus [166,177]. This could also be connected to expanded field work and oceanographic studies that will help to better understand ocean worlds. By better understanding the Earth’s ocean and microbes within different sites (e.g., Lost City [154,155,156]), we can better understand extremophiles and characterize different metabolisms. For this, astrobiologists should connect with oceanographers and Earth scientists.

In addition, the testing of flight-ready instrument techniques in analog chemical reactions as well as field biological samples will provide a database of knowledge on what the instrument is able to detect and how different chemistries can be identified with the instruments. This could show where different instruments could be particularly useful as well as inform the limitations of different instruments for life detection. The use of flight instrument analogs would be especially helpful in this regard. This is especially critical for the instruments of Europa Clipper [7,8] including MASPEX [178] and SUDA [179]. The testing and development of other instruments for flight would also improve the science return of such a mission. Exploring future instruments during development will be critical as well.

## 6. Conclusions

Europa and Enceladus are ocean worlds with probable habitable conditions and organic material, making them prime locations for future spaceflight missions to search for life. In addition, significant work has been conducted to constrain the ocean conditions of these worlds with the limited spaceflight data returned. Based on their ocean conditions, there are a variety of hypothetical metabolisms that could be observed on these worlds if they harbor life. The plume of Enceladus provides a direct look into the chemical composition of the ocean and leads many scientists to believe methanogenesis is possible. Europa, with a much different radiation environment and hypothesized mass transport between the surface and subsurface, is considered to be able to house methane oxidation and sulfate reduction. Field work in extreme environments, such as alkaline hydrothermal vents or serpentinizing systems, additionally would further constrain the kinds of life that could inhabit Enceladus and Europa. Experimental and modeled studies can help to constrain environmental conditions and identify possible chemistries on these worlds, which are difficult to access. One way to make these studies more useful is to analyze the experimental results with mission-relevant techniques (e.g., mass spectrometry, flight instrument analogs) in order to correlate the results to any mission data obtained. As spacecraft data are limited, experimental and modeled studies should be prioritized to understand these worlds. Ultimately, the development of life detection missions to Enceladus and Europa are required to fully address what metabolisms could be present in these oceans.

## Figures and Tables

**Table 1:** Predicted condition comparison between Enceladus and Europa based on modeling and spacecraft measurements. For the molecules and ions, this is a non-exhaustive list.

Condition	Enceladus	Europa
pH	Basic (8–11) [27,28]	Most likely acidic (~4–6), but not well constrained [29,30]
Pressure	1.5–10 MPa [31,32]	110–260 MPa [30,33,34]
Ice shell thickness	5–25 km (excluding south pole terrain) [31,35,36]	5–100 km [37,38,39]
Notable species	H_2_, CO_2_, NH_3_, CH_4,_ organics, phosphates [11,12,13,14,40,41]	Mg^2+^, SO_4_^2−^, CH_4_, NH_3_ [29,42,43,44,45,46,47,48,49,50]

**Table 2:** Recommendations for future work.

Recommendation	Future Work	References
Missions to carry out further characterization of Europa and Enceladus	Data from JUICE, Europa Clipper; proposed Enceladus New Frontiers 5/6 missions, Enceladus Orbilander	[1,7,8,9,10,164]
Abiotic studies	Explore abiotic generation of end member metabolic products, especially under relevant geological conditions	[165,166,167]
Exploration of biotic and abiotic samples on mission-relevant techniques	Test both abiotic and biotic samples on mission-relevant instruments for future ocean worlds missions	e.g., [1,7,8,9,10,164,168,169,170,171,172,173,174,175]
Expanded field work; identifying assumptions on field work	Characterize ocean world-relevant field sites and collect/characterize microbes; understand assumptions and differences of field sites	[154,155,156]

## Data Availability

Not Applicable.
